# Draft Genome Sequence of *Saccharopolyspora rectivirgula*

**DOI:** 10.1128/genomeA.01117-13

**Published:** 2014-01-09

**Authors:** B. M. Fredrik Pettersson, P. R. Krishna Behra, Satyam Manduva, Sarbashis Das, Santanu Dasgupta, Alok Bhattacharya, Leif A. Kirsebom

**Affiliations:** Department of Cell and Molecular Biology, Uppsala University, Uppsala, Swedena; School of Computational and Integrative Sciences, Jawaharlal Nehru University, New Delhi, Indiab; Schools of Life Sciences, Jawaharlal Nehru University, New Delhi, Indiac

## Abstract

We have sequenced the genome of *Saccharopolyspora rectivirgula*, the causative agent of farmer’s lung disease. The draft genome consists of 182 contigs totaling 3,977,051 bp, with a GC content of 68.9%.

## GENOME ANNOUNCEMENT

*Saccharopolyspora rectivirgula* is a Gram-positive thermophilic sporulating actinomycete (1) that causes farmer’s lung disease (FLD), a type of hypersensitivity pneumonitis (2). FLD can develop into a chronic disease and lead to irreversible lung damage and even death (3). *S. rectivirgula* is often found in high concentrations in the air in barns where wet hay is stored (4). Wet hay may reach temperatures of 55 to 60°C, which promotes growth of *S. rectivirgula* (5). Hay can generate dust containing *S. rectivirgula*, which can lead to FLD after inhalation.

Only 1 to 15% of farmers exposed to *S. rectivirgula* develop FLD (6–8). There seems to be a genetic component protecting against the hyperreactive allergic response that is associated with FLD (6, 9, 10). Interestingly, smokers seem to have a lower incidence of the disease (2, 11). By use of a mouse model, it was shown that nicotine reduced the allergic response and lung damage (11). In contrast, other studies have shown that the allergic response gets stronger after a recent viral infection (12, 13).

The only efficient long-term treatment of FLD is removal of the antigen (6, 9). For the individual, this may have large economic and social consequences. Knowing the genome sequence of *S. rectivirgula* may provide a better understanding of the cause of the hyperreactive allergic reaction, which can be used to develop better tools to monitor and detect this working environmental hazard and lead to new methods of treatment.

The *S. rectivirgula* type strain DSM 43747 was obtained from and grown under conditions recommended by the Deutsche Sammlung von Mikroorganismen und Zellkulturen in Germany. Genomic DNA was isolated by lysis using bead beating in equal volumes DNAzol (Invitrogen) and Tris-EDTA (TE) buffer (10 mM Tris [pH 7.9] and 1 mM EDTA) using conditions previously described (14), and the pellet was resuspended in TE buffer. The DNA was treated with RNase A and proteinase K according to standard protocols. Whole-genome sequencing of *S. rectivirgula* was performed at the SNP&SEQ Technology Platform of Uppsala University on a HiSeq2000 (Illumina) platform. A total of 9.3 million paired-end reads were generated, with an average read length of 100 nucleotides. Assembly of the reads was done using the A5 assembly pipeline (15). The reads were assembled into 182 contigs making up a total genome size of 3,977,051 bp with a GC content of 68.9% and an *N*_50_ value of 52,597. The average coverage was 469×. The contigs were annotated using the RAST server (16). This analysis resulted in 3,840 predicted protein-coding genes, 50 tRNA genes, and 2 ribosomal RNA operons. Interestingly, the *S. rectivirgula* genome is considerably smaller than those of the previously sequenced *Saccharopolyspora erythraea* (8.21 Mb) (17) and *Saccharopolyspora spinosa* (8.58 Mb) (18). By use of PanOCT v 1.9 (19), it was predicted that only 1,951 genes are shared among all three genomes and 1,467 genes are present only in *S. rectivirgula*.

### Nucleotide sequence accession numbers.

This whole-genome shotgun project has been deposited in DDBJ/EMBL/GenBank under the accession no. AYJW00000000. The version described in this paper is the first version, AYJW01000000.
